# A Comparative Study on the Phenolic Composition and Biological Activities of *Morus alba* L. Commercial Samples

**DOI:** 10.3390/molecules24173082

**Published:** 2019-08-25

**Authors:** Milena Polumackanycz, Tomasz Sledzinski, Elzbieta Goyke, Marek Wesolowski, Agnieszka Viapiana

**Affiliations:** 1 Department of Analytical Chemistry, Medical University of Gdansk, 80-416 Gdansk, Poland; 2 Department of Pharmaceutical Biochemistry, Medical University of Gdansk, 80-211 Gdansk, Poland; 3 Department of Biochemistry, Medical University of Gdansk, 80-211 Gdansk, Poland

**Keywords:** *Morus alba* L., phenolic composition, antioxidant activity, acetylcholinesterase inhibitory

## Abstract

*Morus alba* L. (white mulberry) has been commonly used as a functional food and for medicinal purposes. Hence, the aim of the study was to compare the phenolic profile of white mulberry commercial samples in relation to their antioxidant potential and acetylcholinesterase (AChE) inhibitory activity. It is of interest to determine whether herbal products originating from different commercial sources differ in their phenolic profiles. For this purpose, a simple and rapid high-performance liquid chromatography (HPLC) method was used for the separation and determination of ten major phenolic compounds. Total phenolic (TPC), total flavonoid (TFC), and total phenolic acid contents (TPAC), as well as l(+)-ascorbic acid (ASA) contents, were determined. The antioxidant potential was assessed by DPPH (2,2-diphenyl-1-picrylhydrazyl radical) scavenging activity and ferric-reducing/antioxidant power (FRAP) assay, while the AChE inhibitory activity was determined by the Ellman assay for water extracts. The study revealed that excluding two herbal products containing fruits and a sample containing leaves of white mulberry, yerba mate and lemon, the remaining samples were generally consistent in terms of phenolic composition as well as antioxidant potential and AChE inhibitory activity. This reflects the health-promoting properties of the samples under study. Moreover, the results showed that the water extracts of white mulberry were richer in phenolic compounds and presented higher antioxidant activity than the hydromethanolic extracts. However, the water extracts showed low inhibitory activity against AChE. Moreover, the correlation analysis indicated a high positive relationship between phenolic composition and antioxidant activity in extracts of white mulberry. Overall, the obtained results may be useful in the evaluation of new dietary supplements and food products. The water extracts of white mulberry could be used for antioxidant purposes, while the hydromethanolic extracts could be incorporated in antioxidant formulations.

## 1. Introduction

White mulberry (*Morus alba* L.) of the Moraceae family is native to Asia but is widely cultivated throughout Africa, Europe, and North and South America [1]. Leaves, fruits, barks, and branches of white mulberry are traditionally used in the treatment of joints, hyperglycemia, dyslipidemia, and hypertension [2,3]. In Chinese medicine, they have long been used to treat fevers, improve eyesight, protect the liver, modulate dendritic cell maturation, and strengthen joints [4,5]. Leaves have also been applied in antibacterial [1,6], anti-diabetic [6,7], antioxidant [6,7,8,9,10], and anti-obesity [11] treatments. In Japan and Korea, patients with diabetes consume mulberry leaves as an anti-hyperglycemic supplement, while in east and southeast Asia, the drinking of mulberry tea, which is richer in γ-aminobutyric acid than green tea, is very popular. In many countries like Greece or Turkey, *Morus alba* and other mulberries are grown for their fruits that have application in some foodstuffs. Mulberry fruits are consumed in both fresh and processed forms, such as juice, syrups, jams, natural dyes, beverages, or dehydrated fruits [12]. Moreover, the fruits, just like leaves, exhibit a variety of biological activities, including anti-obesity, anti-thrombotic, or anti-inflammatory effects [8]. A comprehensive overview of the pharmacological activities of mulberry fruits was reported by Zhang et al. [13].

Recent studies have shown the nutritional value and benefits associated with the consumption of mulberry. Phytochemical studies have identified terpenoids (e.g., linalool, citral, linalyl acetate, and terpinyl acetate), alkaloids including 1-deoxynojirimycin (the most potent glycosidase inhibitor that decreases blood sugar levels), stilbenoids (e.g., resveratrol, piceatannol, rhapontigenin, astringin, pterostilbene, piceid, rhaponticin, and vitisin A), flavonoids (e.g., quercetin, rutin, isoquercetin, cyaniding 3-rutinoside, cyaniding 3-glucoside, and 3-(-malonyglucoside)), phenolic acids (e.g., gallic, protocatechuic, *p*-hydroxybenzoic, vanillic, chlorogenic, syringic, *p*-coumaric, ferulic and *m*-coumaric), and coumarins in *Morus alba* [14,15,16,17,18,19]. Among these compounds, rutin, quercetin, and apigenin are the main bioactive constituents in white mulberry leaves.

Because of its role in the hydrolysis of the neurotransmitter acetylcholine (ACh), the acetylcholinesterase enzyme (AChE) is a target for rational drug design as well as for the discovery of mechanism-based inhibitors. AChE inhibitors offer an effective approach to treat the cognitive symptoms of Alzheimer’s disease [20]; they might also have other therapeutic applications, i.e., in the treatment of Parkinson’s disease, ataxia, myasthenia gravis, and dementia [21]. They are also used in several synthetic medicines, e.g., donepezil or tacrine; natural product-based rivastigmine is used in the treatment of cognitive dysfunction and memory loss associated with Alzheimer’s disease. The compounds have been reported to have some adverse effects, too, including gastrointestinal disturbances and problems associated with bioavailability. For this reason, there is considerable interest in finding better AChE inhibitors from natural resources.

Natural products have already proven to be promising sources of useful acetylcholinesterase (AChE) inhibitors. There has been a lot of research on the biological effect of plants traditionally used either in infusions or in traditional remedies as acetylcholinesterase inhibitors in vitro, and also as memory enhancers in vivo. One of the main benefits is that they have a low toxicity compared to pharmaceutical agents. The majority of AChE inhibitors belong to the alkaloid group, including isoquinoline or indole, as well as steroidal alkaloids, flavonoids, terpenoids, and other phenolic compounds [22].

Therefore, the aim of this study was to compare the phenolic profile of white mulberry (*Morus alba* L.) commercial samples using two extraction solvents, hydromethanol and water, in relation to their antioxidant property and acetylcholinesterase (AChE) inhibitory activity. It is of interest to determine whether herbal products originating from different commercial sources will differ in their phenolic profile. For this reason, the total content of phenolics, flavonoids, phenolic acids and l(+)-ascorbic acid, and the content of individual phenolic acids and flavonoids were quantified in hydromethanolic and water extracts were obtained from the samples under study. The antioxidant activity of white mulberry extracts was evaluated using DPPH free radical scavenging and ferric-reducing/antioxidant power (FRAP) assays. The acetylcholinesterase (AChE) inhibitory activity was determined for water extracts because of their common use as herbal teas. To date, data on the AChE inhibitory activity of white mulberry are missing in the literature.

## 2. Results and Discussion

### 2.1. Validation of HPLC-UV/Vis Method

Method validation (Table 1) was performed by evaluating the following parameters: linearity, limits of detection (LOD) and quantification (LOQ), intra- and inter-day precision, recovery, and stability. A hydromethanol extract of white mulberry sample 2 was used for validation purposes. Linearity was established by plotting the peak area vs. concentration of each determined compound. The LOD and LOQ were calculated in µg/mL in accordance with the following equations: LOD = 3.3S_xy_/b and LOQ = 10S_xy_/b, where S_xy_ is the standard deviation of the response, and b is the slope of the calibration curve. As can be observed, good linearity was found over the determined ranges for all analytes, with correlation coefficient (r) values significantly higher than 0.984. The values of LODs and LOQs were less than 5.86 and 14.68 µg/mL, respectively. Intra-day precision was validated with a standard solution of assayed phenolic compounds three times within one day, while inter-day precision was validated with the same standard solution over three consecutive days.

Consequently, the precision was acceptable, and coefficient of variation (CV) values ranged from 0.6% to 1.6% and 1.0% to 3.6% for intra- and inter-day variations, respectively. To assess the recovery, known quantities of the standard solutions were added to the previously analyzed hydromethanol extract. After extraction, a sample was processed and quantified according to the procedure described in Section 3.4.5. HPLC analysis. The mean recovery was found to be in a satisfactory range, 93.8–97.5%, with a relative standard deviation (RSD) less than 4.1%. The peak areas and retention times of ten phenolic compounds were analyzed every eight hours within 48 h for the stability test and they were found to be quite stable, while retention CV was lower than 1.5% for peak area and 0.3% for retention time.

### 2.2. Total Phenolic, Flavonoid, Phenolic Acid and l(+)-Ascorbic Acid Contents

Since polyphenols are considered to be amongst the most biologically active constituents, contributing greatly to antioxidant activity, total phenolic (TPC), total flavonoid (TFC), total phenolic acid (TPAC) and l(+)-ascorbic acid (ASA) contents in commercial samples of white mulberry were determined. The results shown in Table 2 revealed that water extracts were significantly richer (*p* < 0.05) in TPC, TFC, TPAC, and ASA than hydromethanolic extracts. Thabti et al. [15] determined TPCs and TFCs in leaves and stem barks of white mulberry, and also found that water extracts are richer in TPC and TFC than hydromethanolic extracts. Despite the fact that many authors found methanol as the most effective solvent for phenolics extraction, the plant material contains a wide variety of bioactive constituents and, thus, the choice of optimal solvent for their extraction depends inter alia on the plant genus [23]. Moreover, the yield of extraction depends on many factors; the solvent type, extraction time, temperature and composition of a sample are the most important of them. The higher levels of phenolic compounds in water extracts of white mulberry obtained in this study may be due to the use of hot water for extraction. In water extracts, the mean TPCs were at a level of 4.67 mg gallic acid equivalents (GAE)/g dry weight (DW), while in hydromethanolic extracts, they were several times lower. The TPCs obtained in this study differ from those in the relevant literature. Kim et al. [24] found higher TPCs in methanolic extracts of mulberry, in the range from 28.2 to 55.4 mg GAE/g DW. These results are consistent with those found by Zou et al. [25]—from 30.4 to 44.7 mg GAE/g DW. Jia et al. [26] found lower values of TPC in mulberry leaves—from 9.8 to 29.6 mg rutin/g DW. Bazylak et al. [27] also detected TPCs at a low level of 600 mg caffeic acid equivalent (CAE)/100 g DW in water extracts of white mulberry leaves from Poland, China and Bulgaria, while Memon et al. [14] determined TPCs in white mulberry leaves at a level of 8.33 mmol GAE/100 g DW. Such literature data revealed that the efficiency of phenolic extraction depends on the solvent type, extraction method, and drying process used for the plant material [28].

The biological activity of many plant materials depends on flavonoids; hence, studies on the variation in their contents are important [29]. The TFCs ranged between 26.37 and 96.38 µg quercetin equivalent (QE)/g DW in the hydromethanolic extracts and from 0.191 to 0.607 mg QE/g DW in the water extracts of white mulberry commercial samples. A higher level of TFCs in water extracts of white mulberry leaves (302.70 mg CAE/100 g DW) was reported by Bazylak et al. [27]. Jia et al. [26] and Chen et al. [17] also detected higher values of TFCs—from 11.7 to 26.6 mg rutin/g DW and from 18.75 to 62.01 mg rutin/g—while Thabti et al. [1] obtained TFCs at a level of 283.13 mg rutin/100 g DW for hydromethanolic extracts and 717 mg rutin/100 g DW for water extracts of white mulberry leaves.

Several studies have reported the inhibitory effect of phenolic acids on the growth of pathogens and cancer cells [29]. The TPACs varied from 12.88 to 88.11 µg CAE/g DW for hydromethanolic extracts and samples 5, 11 and 12 were the richest. In the case of water extracts, the level of TPACs was several times higher than that in hydromethanolic extracts, ranging from 127.82 to 573.04 µg CAE/g DW. Ascorbic acid is essential for the normal functioning of living cells and many enzymatic reactions in humans. The ASA content varied from 0.124 to 0.967 mg ASA/g DW and from 1.17 to 2.23 mg ASA/g DW for hydromethanolic and water extracts, respectively. Unfortunately, the obtained results of TPACs and ASA cannot be compared with previous studies since such analyses were not previously performed.

### 2.3. Quantification of Phenolic Compounds

**Figure 1:**
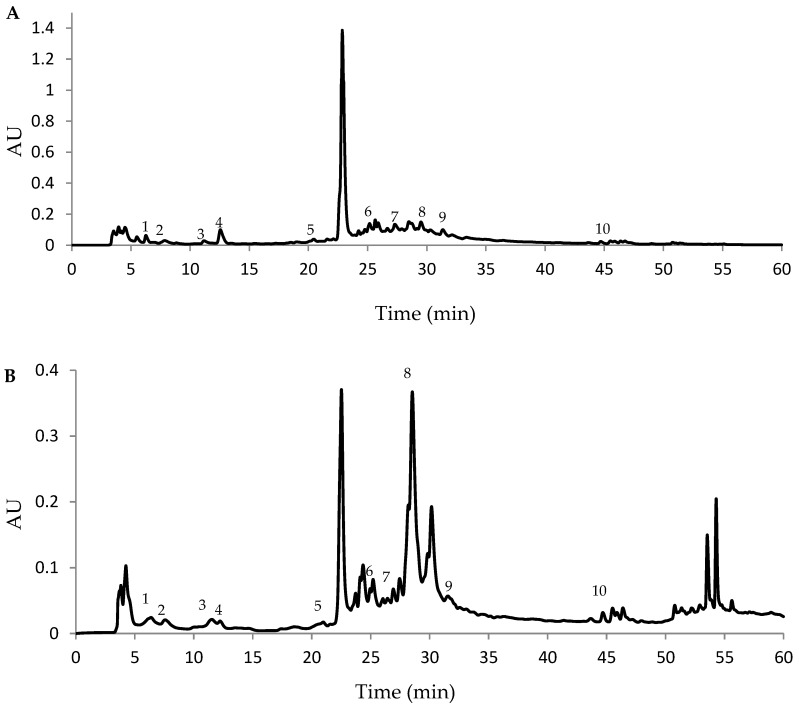
HPLC phenolic profile of (**A**) hydromethanolic and (**B**) water extracts prepared from commercial *Morus alba* L. (sample 2). The retention times (min) for quantified compounds were as follows: 5.88 (gallic acid, peak 1), 7.25 (caffeic acid, peak 2), 10.74 (*p*-coumaric acid, peak 3), 11.97 (chlorogenic acid, peak 4), 20.18 (vanillic acid, peak 5), 24.63 (ferulic acid, peak 6), 26.68 (rosmarinic acid, peak 7), 28.74 (rutin, peak 8), 30.90 (quercetin, peak 9) and 44.16 (apigenine, peak 10).

To determine the phenolic acids and flavonoids in hydromethanolic and water extracts of commercially available white mulberry samples, a simple and reliable HPLC procedure was developed. Typical HPLC chromatograms are shown in Figure 1. The ANOVA test showed no statistically significant differences among the white mulberry samples based on the contents of phenolic constituents. As indicated by the data summarized in Table 3, the concentrations of phenolic acids and flavonoids of white mulberry commercial samples represent the following order: chlorogenic acid > rutin > quercetin > apigenin > rosmarinic acid > gallic acid > ferulic acid > caffeic acid > *p*-coumaric acid > vanillic acid for hydromethanolic extracts, and rutin > chlorogenic acid > quercetin > gallic acid > rosmarinic acid > apigenin > ferulic acid > caffeic acid > *p*-coumaric acid > vanillic acid for water extracts. In the case of hydromethanolic extracts, samples 3, 8 and 18 are the richest in analyzed phenolic compounds, while samples 6, 9, 10, 13 and 16 are the poorest. For water extracts, samples 4, 6 and 18 are the richest, while samples 7, 11 and 13 are the poorest in phenolic constituents.

Generally, sample 18, regardless of the type of the extract, is the richest in phenolic compounds, especially in chlorogenic and caffeic acids, and quercetin. This fact may be due to the presence of other ingredients in the sample besides the white mulberry leaves, such as yerba mate and lemon. It is well known that combining different herbs consisting of various bioactive constituents results in either synergistic or antagonistic biological effects. In the case of sample 18, the mixture of two and more herbs could improve the antioxidant potential of herbal tea products. Sample 6, for instance, is rich or poor in phenolic compounds, depending on the type of the extract. One-way variance analysis (ANOVA) indicated that there are also no relationships between composition, confection or place of origin and phenolic compound concentrations.

The mean concentration of rutin was at a level of 6.22 mg/g DW for hydromethanolic extracts and 28.08 mg/g DW for water extracts of white mulberry commercial samples. The results for hydromethanolic extracts are consistent with those obtained by Kutsabe et al. [30] for hydroethanolic extracts (573 mg/100 g DW). Zou et al. [24] also analyzed hydroethanolic extracts and found a lower level of rutin—from 0.4 to 1.2 mg/g DW—while Kim et al. [24] determined 3.20 mg rutin/g DW in methanolic extracts of Korean white mulberry. Memon et al. [14] used three types of extraction to determine phenolic compounds in hydromethanolic extracts of white and black mulberries, originating from Pakistan and found vanillic acid on a similar level to that found in this study.

The chlorogenic, *p*-coumaric, ferulic and gallic acids levels were found to be higher than those found by Memon et al. [14]. Flaczyk et al. [31] also determined phenolic compounds in white mulberry leaves from Poland and found ferulic acid at the same level. In the case of gallic acid, the results obtained in this study were higher, while for vanillic, caffeic and *p*-coumaric acids, the results obtained were slightly lower on comparing with the results obtained by Flaczyk et al. [31]. This could be explained by the differences in the growing conditions in different regions [32]. Moreover, a literature screening also revealed that the efficiency of phenolic extraction depends on the extraction method, solvent type and drying process used for the plant material [28].

### 2.4. Antioxidant Activity

The antioxidant activity is an important parameter for establishing the health benefits of food products [30]. Several tests are used to estimate this activity, e.g., DPPH and FRAP assays, which are complementary to the same degree and are recommended as rapid, simple, low-cost and reproducible tools for measuring the antioxidant potential of plant extracts [33].

The antioxidant potential of 18 commercial samples of white mulberry is shown in Table 4. In general, the hydromethanolic extracts gave lower DPPH values and higher FRAP values than water extracts. An antioxidant activity-based comparison of the samples rich in phenolic compounds might give different conclusions depending on the choice of assay due to different structure-activity relationships between polyphenol substituents and assay results [34]. Furthermore, the antioxidant activity may be a result of the presence of different constituents, not determined in this study, which are present in white mulberry extracts. In the DPPH assay, the values ranged from 23.38 to 35.74 mg trolox (TE)/100 g DW for hydromethanolic extracts and from 52.41 to 98.82 mg TE/100 g DW for water extracts. The values of FRAP for hydromethanolic and water extracts were from 18.10 to 37.85 mmol Fe^2+^/g DW and from 5.96 to 21.21 mmol Fe^2+^/g DW, respectively. In hydromethanolic extracts, the highest DPPH values were determined in samples 9 and 10, which contained fruits of white mulberry, while the values of FRAP in these samples were of the lowest levels, below 20 mmol Fe^2+^/g DW. The low values of FRAP were also found in samples 5 and 13. Among the water extracts, samples 4, 10 and 14 were characterized by the highest values of DPPH (above 80 mg TE/100 g), while in sample 1, the lowest levels of DPPH and FRAP assays were observed. Sample 18, which besides the white mulberry leaves also contained yerba mate and lemon, was characterized by high FRAP assays both in hydromethanolic and water extracts. The obtained results revealed that the water extracts had higher antioxidant activities than the hydromethanolic extracts of white mulberry samples (ANOVA). The results of the DPPH assays are similar to those obtained by Memon et al. [14] for *Morus alba* leaves (65.99 µmol quercetin equivalent/100 g). Butkhup et al. [35] assessed the antioxidant activity of ethanolic extracts of *Morus alba* L. fruits on levels ranging from 241.83 to 1194.67 µg/mL (DPPH radical concentration by 50%). For the FRAP assay, no data for the mulberry samples have been previously reported.

Antioxidant activity of commercial samples of white mulberry was lower than that of the standards. DPPH values of the extracts were several times lower than those for gallic acid (1426 mg/100 g), caffeic acid (457 mg/100 g), rutin (258 mg/100 g) and ascorbic acid (763 mg/100 g). The same applies to the FRAP values which are also lower than those of the standards: gallic acid (58.80 mmol Fe^2+^/g), caffeic acid (71.20 mmol Fe^2+^/g), rutin (48 mmol Fe^2+^/g) and ascorbic acid (42 mmol Fe^2+^/g). The obtained results for the standards demonstrate the difference in antioxidant activities of the reference antioxidants and selected phenolic and flavonoid compounds in different assays. This may be due to the fact that the different antioxidant capacity determining methods have different specificities for different solvents, reagents, pH conditions, or hydrophilic/hydrophobic substances. Moreover, molecular size and the number and type of the functional groups of the phenolic and flavonoid compounds may be important.

### 2.5. Acetylcholinesterase Inhibitory Activity

The AChE inhibitory activity of the water extracts of *Morus alba* leaves is presented in Table 4. It was found that all of the extracts had an acetylcholinesterase (AChE) inhibitory activity below 50%. It confirms that the water extracts of *Morus alba* leaf commercial samples did not show any significant AChE inhibitory activity. Among the analyzed samples, four of them (samples 5, 10, 11 and 13) have got the lowest values of AChE inhibitory activity. This could be explained by a lower content of polyphenolic compounds [36,37] and lower antioxidant activity among the analyzed water extracts [38]. The standards (gallic acid, caffeic acid, rutin, and ascorbic acid) also did not show any significant AChE inhibitory activity. According to relevant literature, the leaves of *Morus alba* have been demonstrated to exert anti-Alzheimer’s disease activities through the inhibition of amyloid beta-peptide (1–42) fibril formation and the attenuation of amyloid beta-peptide (1–42)-induced neurotoxicity [39]. Kuk et al. [40] demonstrated that the root bark of *Morus alba* has the most potent inhibitory activity against AChE (IC50 = 48.4 µg/mL) among fruits, branches, and leaves. Moreover, the methanolic extracts of *Morus alba* root bark and its different solvent-soluble fractions (ethyl acetate, butanoic, aqueous, chloroform) were investigated for the inhibition of AChE. The results obtained by Kuk et al. [40] showed that the fraction of ethyl acetate had the most potent inhibitory activity. These variations are possibly due to differences in type of the extracts and their chemical composition. Moreover, population differences of the same species also determine the anti-acetylcholinesterase activity [36].

### 2.6. Discrimination of White Mulberry Commercial Samples

**Figure 2:**
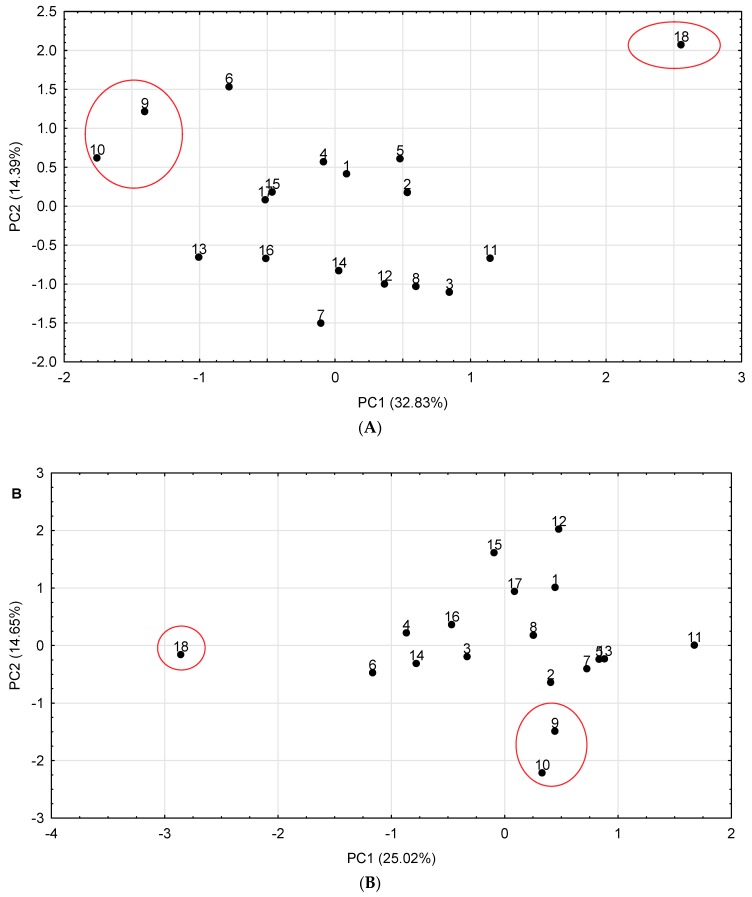
The principal component analysis score plot of the first two components for (**A**) hydromethanolic extracts and (**B**) water extracts of *Morus alba* L.

To show whether or not particular commercial samples of *Morus alba* L. differ widely in phenolic profile and antioxidant activity, principal component analysis (PCA) was used. A graphical illustration of PCA calculations is shown in Figure 2. It indicates that according to the first principal component (PCl) axis, *Morus alba* L. samples are gathered into two sectors. Sample 18 (contained leaves of white mulberry, yerba mate and lemon, Table 5) can be found on the right part of the scatterplot for hydromethanolic extracts, and on the left for water extracts. This sample was rich in phenolic compounds (Table 2 and Table 3) and has high antioxidant activity (Table 4). The remaining samples of white mulberry can be grouped into two clusters in the central sector of the PCA plot. Samples 9 and 10, containing fruits, are separated from the other commercial samples due to their lowest content of phenolic compounds and the lowest antioxidant activity.

### 2.7. Inter-Correlation Among Analytes and Biological Activity

The correlation analysis showed 22 statistically significant correlations (*p* < 0.05) for hydromethanolic extracts. The highest correlations (r > 0.7) were obtained between total flavonoid contents and chlorogenic acid; caffeic acid and ascorbic acid; and between total phenolic contents and ascorbic acid. In water extracts, 13 statistically significant correlations were found and the highest correlation (r > 0.7) was obtained between ferulic acid and AChE inhibitory activity. Moreover, the correlation of antioxidant activities and total phenolic contents, ferulic acid, quercetin, ascorbic acid, chlorogenic acid in hydromethanolic extracts, and total flavonoid contents in water extracts of mulberry samples were found to be moderately positive. The obtained results suggest the crucial role of these phenolic compounds as antioxidant constituents in the mulberry extracts. The correlation between TFC and DPPH in hydromethanolic and water extracts of mulberry was moderately negative, at −0.54 and −0.57, respectively. The cases where values of the Pearson’s correlation coefficients were below 0.5 suggest that the constituents that occur separately in the extracts could not be responsible for the antioxidant properties.

Memon et al. [14] used three different extractions methods (ultrasonic-assisted extraction, magnetic stirring, and homogenization) for the determination of phenolic acid profile, sugar content and the antioxidant activities of the leaves and fruits of three mulberry species grown in Pakistan. These researchers observed a high positive correlation (r = 0.803) between TPC and DPPH scavenging activity extracted only by stirring. Iqbal et al. [41] studied the proximate composition and antioxidant activities of leaves from three varieties of mulberry and found very weak positive correlations between ASA and DPPH, ASA and FRAP, and TPC and DPPH, and a very strong negative correlation between TPC and FRAP (r = −0.932). These results confirm the contribution of many other components such as vitamins, anthocyanins and carotenoids to the antioxidant activity exhibited by the sample extracts.

## 3. Materials and Methods

### 3.1. White Mulberry Samples

Eighteen commercial samples of *Morus alba* L. were purchased in local supermarkets and a pharmacy (Table 5). The majority of samples came from Poland; most samples contained leaves of white mulberry in the form of tea bags. Only two samples contained fruits and one sample contained herbs; seven samples were in a loose form. The samples were pulverized in a water-cooled Knifetec 1095 grinder (Foss Tecator, Höganäs, Sweden) and the homogenized samples were stored in a light-proof desiccator.

### 3.2. Chemicals and Instruments

Standards of phenolic acids—gallic, vanillic, caffeic, chlorogenic, ferulic, *p-*coumaric, rosmarinic —and flavonoids—rutin, quercetin and apigenin—were purchased from ChromaDex (California, USA). Acetonitrile and HPLC-grade methanol were purchased from J.T. Baker (Phillipsbusg, USA) and POCh (Gliwice, Poland), respectively. Analytical-grade methanol, ethanol and acetic acid were purchased from POCh (Gliwice, Poland). Redistilled water was prepared by the triple distillation of water in a Destamat bi-18 system (Heraeus Quarzglas, Hanau, Germany).

The separation and quantitation of phenolic compounds were performed using a HPLC Merck-Hitachi LaChrome device (Darmstadt, Germany), equipped with a L-7420 UV–Vis detector, a L-7200 autosampler and a L-7360 thermostat. Chromatographic data were collected using a D-7000 HPLC System Manager, ver. 3.1 (Merck-Hitachi, Darmstadt, Germany). The method developed for the quantitation of ten phenolic compounds was validated by linear range, limit of detection (LOD), limit of quantitation (LOQ), precision and recovery in accordance with the procedure described above [42].

Total phenolic, flavonoid, phenolic acid, l(+)-ascorbic acid contents and antioxidant activities were determined using a Metertech UV/Vis spectrophotometer (Nankang, Taiwan). Absorbance was measured with the use of a 10 mm quartz cuvette at a suitable wavelength: at 280 nm for gallic, vanillic and rosmarinic acids, at 320 nm for caffeic, chlorogenic, ferulic and *p-*coumaric acid, and 370 nm for rutin, quercetin and apigenin.

### 3.3. Sample Preparation

In this study, hydromethanolic and water extracts of white mulberry were prepared. For the hydromethanolic extracts, each sample (1.0 g) was sonicated with 4 mL of a mixture of methanol and water (80:20, *v/v*) for 10 min at 20 °C using an ultrasonic bath (Emag, Salach, Germany). The suspension was centrifuged in an EBA-20S centrifuge (Hettich, Tuttlingen, Germany) for 5 min at 8000 rpm and the supernatant transferred into a 20 mL volumetric flask. This procedure was repeated twice, after which the extracts obtained were combined and diluted by up to 20 mL with a mixture of methanol and water (80:20, *v*/*v*).

For the water extracts, each sample of mulberry (1.0 g) was added to 200 mL of boiling water at 100 °C. Then the mixture was left to stand at room temperature for 10 min and filtered under reduced pressure.

Before HPLC analysis, hydromethanolic and water extracts of white mulberry were filtered through a 0.45 μm nylon filter film (Mecherey, Nagel, Germany) and 20 μL of the filtrate was injected into the HPLC system.

### 3.4. Determination of Phytochemical Compounds

#### 3.4.1. Total Phenolic Content

TPC of the white mulberry extracts was determined using the Folin–Ciocalteu method, as previously described by Singleton and Rossi [43] with some modifications. Briefly, an appropriate amount of the extract was mixed with 0.2 mL of Folin–Ciocalteu reagent. The mixture was left to settle for 3 min; then, 2 mL of 7% (*w*/*v*) Na_2_CO_3_ solution was added and incubated in the dark at room temperature for 1 h. The absorbance of the mixture was measured at 760 nm. A calibration curve was prepared with gallic acid to obtain a correlation between sample absorbance and standard concentration (linearity range: 20–70 µg/mL, r = 0.996). The TPC of the extracts was expressed in mg gallic acid equivalents per gram dry weight of sample (mg GAE/g DW).

#### 3.4.2. Total Flavonoid Content

TFC of the white mulberry extracts was determined with the method described in the European Pharmacopoeia [44] method with some modifications. An appropriate amount of the extract was mixed with 0.1 mL of 5% (*w*/*v*) AlCl_3_ solution. The mixture was incubated for 30 min in the dark at room temperature and the absorbance was measured at 430 nm. Quercetin was used as reference to plot the standard curve (linearity range: 22–66 µg/mL, r = 0.998). The TFC was expressed as µg of quercetin equivalent per gram dry weight of sample for hydromethanolic extracts (µg QE/g DW) and as mg of quercetin equivalent per gram dry weight of sample for water extracts (mg QE/g DW).

#### 3.4.3. Total Phenolic Acid Content

The procedure described in the Polish Pharmacopoeia VI [45] was used for TPAC determination with Arnov’s reagent. An appropriate amount of the extract was mixed with 0.2 mL of 0.5 N HCl, 0.2 mL of Arnov’s reagent and 0.2 mL of 1 M NaOH. Absorbance was measured at 490 nm and caffeic acid was used as a reference to plot the standard curve (linearity range: 2.40–16.8 µg/mL, r = 0.999). The results were expressed in µg of caffeic acid equivalent per gram dry weight of sample (µg CAE/g DW).

#### 3.4.4. l(+)-Ascorbic Acid Content

The Abdelmageed method [46] was used for ASA determination. An appropriate amount of the extract was mixed with 0.2 mL of 0.2 M of NaOH, 0.2 mL of 4-chloro-7-nitrobenzofurazane (NBD-Cl) and 1.4 mL of 50% (*v*/*v*) aqueous acetone solution. After 30 min, the absorbance was read at 582 nm, while the calibration curve was prepared with ascorbic acid (linearity range: 8.50–32.50 µg/mL, r = 0.895). The content of l(+)-ascorbic acid of the extract was expressed as mg of ascorbic acid per gram dry weight of sample (mg ASA/g DW).

#### 3.4.5. HPLC Analysis

The chromatographic separation and quantitation of the phenolic compounds were performed in a Hypersil Gold C18 column (250 × 4.6 mm, 5 μm particles) (Thermo Scientific, Runcorn, UK), maintained at 40 °C, using a acetonitrile–0.5% acetic acid solution (solvent A) and a water–0.5% acetic acid solution (solvent B) as the mobile phase. The separation was performed at a constant flow rate (1 mL/min) with the following conditions: linear gradient from 2% to 5% of A in 15 min, from 5 to 15% in 5 min, from 15 to 25% in 10 min, from 25 to 35% in 15 min, from 35 to 65% in 5 min and then isocratic elution in 5 min and a linear gradient from 65 to 2% in 5 min. The absorbance was monitored at 280, 320 and 370 nm for benzoic and cinnamic acid derivatives, and flavonoids, respectively.

The identification of the analyte compounds was based on a comparison of the retention time of their standard compounds. Additionally, a selected sample was spiked with the standard compounds and analyzed again. The phenolic compound estimation was made with the use of calibration curves of known concentration (20–200 µg/mL) for each phenolic standard.

### 3.5. Evaluation of Antioxidant Activity

#### 3.5.1. DPPH Scavenging Activity Assay

The DPPH assay was performed in accordance with a modified method of Tuberoso et al. [47]. This method is based on the DPPH free radical scavenging ability. An appropriate amount of the extract was added to 1.6 mL of methanolic DPPH solution (100 μmol/L) and, after 30 min, the absorbance was measured at 517 nm. A calibration curve was prepared with trolox (linearity range: 100–300 mg/mL, r = 0.971). The data obtained were expressed in mg Trolox equivalent per 100 grams dry weight of sample (mg TE/100 g DW).

#### 3.5.2. FRAP Assay

The ferric-reducing/antioxidant power (FRAP) assay was determined using the method proposed by Benzie and Strain [48]. This method is based on the reduction at low pH of ferric-2,4,6-tris(pyridin-2-yl)-1,3,5-triazine, (FeIII-TPTZ) to the ferrous complex [45]. An appropriate amount of extract was mixed with 2.25 mL of FRAP reagent. After incubation at room temperature for 30 min, the absorbance of the reaction mixture was measured at 593 nm. A calibration curve was prepared with ferrous sulphate (linearity range: 83–600 µmol/L, r = 0.992) and the results were expressed in mmol ferrous ion equivalents per gram dry weight of sample (mmol Fe^2+^/g DW).

### 3.6. Determination of AChE Inhibitory Activity

Acetylcholinesterase inhibitory activity was assayed by Ellman’s method, adapted to a 96-well microplate [49]. Briefly, incubation mixtures containing 0.02 U/ml acetyl type IV-S cholinesterase from electric eel (Sigma-Aldrich), 1.5 mM Ellman’s reagent (DTNB), 1.5 mM acetylthiocholine iodide and 10 mM tris (pH 8.0) were incubated for 10 min with the addition of 20 µL of water or water extracts of Morus alba leaves in a total volume of 200 µL in microplate wells. During incubation, AChE hydrolyses the acetylthiocholine to thiocholine, which in reaction with DTNB produces 2-nitrobenzoate-5-mercaptothiocholine and 5-thio-2-nitrobenzoate, which can be detected by measuring the absorbance at 412 nm. The absorbance at 412 nm was recorded during incubation on a microplate by BioTek Synergy HT microplate reader (BioTek Instruments Winooski, VT, USA), and verified for linearity. The inhibitory activity was calculated as a percentage of the velocities compared to the control assay without extract [50]. The presented data are the average of three replicates.

### 3.7. Data Analysis

The analyses were carried out at least in triplicate. The data were analyzed using the one-way analysis of variance (ANOVA) test, followed by Duncan test. The relationship between mulberry extracts based on the chemical composition, antioxidant activity, and AChE activity was analyzed in a Pearson correlation analysis, while the variation between the mulberry samples was evaluated using principal component analysis (PCA). Statistical data analysis was performed using Statistica 10 software (StatSoft Inc., Tulsa, OK, USA) on the basis of parametric tests with the level of significance of *p* < 0.05.

## 4. Conclusions

*Morus alba* L. is a plant appreciated by consumers and is described as a food with several health benefits. This study on the phenolic profile, antioxidant properties, and AChE inhibitory activity of 18 commercially available samples of white mulberry revealed that excluding two herbal products containing fruits (samples 9 and 10) and sample 18 containing leaves of white mulberry, yerba mate and lemon. The remaining samples are generally consistent in terms of phenolic composition as well as antioxidant potential and AChE inhibitory activity, regardless of the place of origin or confection of the sample studied. This reflects the health-promoting properties of the samples under study. This research also showed the effect of extraction solvents (hydromethanol and water) on the phenolic composition of extracts and efficiency of extraction. Both hydromethanolic and water extracts are good sources of phenolic compounds and revealed high antioxidant activity. However, water extracts were richer in phenolic constituents and were characterized by higher values of TPCs, TFCs, TPACs, and L(+)-ascorbic acid content. Among the quantified plant secondary metabolites, chlorogenic acid, rutin, apigenin, and quercetin were found in the highest concentrations in the extracts, while *p*-coumaric and vanillic acids were in the lowest level. The relationships between analyzed phenolic compounds and the biological activity of their extracts suggest the crucial role of phenolic constituents as antioxidant agents in mulberry extracts. Overall, the study demonstrated the rich phenolic composition and high antioxidant potential of white mulberry accessible to consumers in the form of tea infusion or by incorporating hydromethanolic extracts in antioxidant herbal formulations.

## Figures and Tables

**Table 1:** Validation parameters for the phenolic compounds in *Morus alba* L. (*n* = 3).

Phenolic Compounds	Gallic Acid	Vanillic Acid	Caffeic Acid	Chlorogenic Acid	Ferulic Acid	*p*-Coumaric Acid	Rosmarinic Acid	Rutin	Apigenin	Quercetin
Range (µg/mL)	20–200	20–140	20–140	20–200	20–200	20–200	20–200	20–200	20–200	20–140
r	0.998	0.991	0.989	0.985	0.999	0.997	0.998	0.998	0.983	0.985
LOD(µg/mL)	4.05	3.76	3.76	5.86	3.43	4.17	2.54	2.76	2.87	2.98
LOQ(µg/mL)	13.07	10.88	11.04	14.68	10.12	11.22	6.98	7.43	7.13	9.56
Intra-day precision										
Nominal conc. (µg/mL)	80	80	80	80	80	80	80	80	80	80
Assayed conc. (µg/mL)	78.4	78.2	78.7	77.6	77.3	78.5	78.1	77.8	77.6	79.2
Recovery (%)	98.0	97.7	98.3	97.0	97.8	96.8	97.6	97.2	97.0	99.0
CV (%)	0.6	0.8	1.0	1.3	0.9	1.4	0.7	1.3	1.6	1.4
Inter-day precision										
Nominal conc. (µg/mL)	80	80	80	80	80	80	80	80	80	80
Assayed conc. (µg/mL)	67.6	77.2	74.3	77.0	75.5	71.8	72.5	76.1	73.6	74.6
Recovery (%)	84.7	96.5	92.8	96.2	94.3	89.7	90.6	95.1	92.0	93.2
CV (%)	1.0	1.4	1.8	1.9	2.8	2.0	1.5	2.2	3.6	3.5
Recovery										
Mean	94.8	95.3	96.8	95.7	94.1	93.8	96.5	96.4	95.8	97.5
RSD (%)	2.7	1.8	3.4	3.3	4.1	2.5	3.2	1.6	1.2	3.9

**Table 2:** Total phenolic (TPC), flavonoid (TFC), phenolic acids (TPAC) and ascorbic acid (ASA) contents in commercial samples of *Morus alba* L. (arithmetic mean ± standard deviation).

	Hydromethanolic Extracts				Water Extracts			
	TPC	TFC	TPAC	ASA	TPC	TFC	TPAC	ASA
	(mg GAE/g)	(µg QE/g)	(µg CAE/g)	(mg/g)	(mg GAE/g)	(mg QE/g)	(µg CAE/g)	(mg ASA/g)
1.	0.637^defg^ ± 0.03	63.87^cd^ ± 1.58	32.21^f^ ± 0.58	0.322^de^ ± 0.04	5.45^h^ ± 0.18	0.552^g^ ± 0.01	364.17^c^ ± 7.4	1.17^a^ ± 0.14
2.	0.717^e^ ± 0.05	65.29^cd^ ± 3.35	26.43^ef^ ± 1.03d	0.303^cde^ ± 0.01	4.34^de^ ± 0.27	0.358^c^ ± 0.05	279.00^b^ ± 8.9	1.32^a^ ± 0.21
3.	0.257^a^ ± 0.04	80.61^e^ ± 1.66	21.42^bcd^ ± 0.94	0.273^bcd^ ± 0.03	4.03^cde^ ± 0.21	0.482^ef^ ± 0.02	260.78^b^ ± 9.0	1.28^a^ ± 0.07
4.	0.724^g^ ± 0.05	50.59^b^ ± 8.58	31.51^ef^ ± 7.59	0.459^f^ ± 0.05	4.03^cde^ ± 0.54	0.361^c^ ± 0.04	518.19^e^ ± 4.4	1.48^a^ ± 0.17
5.	0.575^cde^ ± 0.01	71.32^de^ ± 5.60	66.01^i^ ± 7.39	0.631^g^ ± 0.05	7.39^i^ ± 0.17	0.513^fg^ ± 0.01	162.61^a^ ± 6.8	1.99^b^ ± 0.18
6.	0.338^b^ ± 0.03	30.75^a^ ± 6.17	17.86^ab^ ± 0.13	0.363^e^ ± 0.01	3.56^abc^ ± 0.29	0.411^d^ ± 0.03	273.16^b^ ± 6.7	1.28^a^ ± 0.15
7.	0.692^fg^ ± 0.05	79.12^e^ ± 2.10	18.42^abc^ ± 1.39	0.327^de^ ± 0.03	4.13^de^ ± 0.17	0.408^d^ ± 0.01	452.07^d^ ± 4.4	2.05^b^ ± 0.14
8.	0.367^b^ ± 0.06	96.38^f^ ± 9.17	14.07^a^ ± 0.71	0.323^de^ ± 0.01	4.53^ef^ ± 0.07	0.430^d^ ± 0.06	253.35^b^ ± 4.1	1.25^a^ ± 0.03
9.	0.350^b^ ± 0.09	26.96^a^ ± 0.14	12.88^a^ ± 0.21	0.124^a^ ± 0.04	3.38^ab^ ± 0.15	0.234^b^ ± 0.01	134.26^a^ ± 2.4	1.54^a^ ± 0.22
10.	0.299^b^ ± 0.04	26.37^a^ ± 0.29	12.96^a^ ± 0.87	0.220^b^ ± 0.07	3.18^a^ ± 0.17	0.191^a^ ± 0.01	127.82^a^ ± 2.5	2.13^b^ ± 0.52
11.	0.311^b^ ± 0.06	93.34^f^ ± 2.76	59.75^h^ ± 3.40	0.596^g^ ± 0.06	8.30^j^ ± 0.24	0.494^f^ ± 0.02	368.26^c^ ± 7.9	2.02^b^ ± 0.13
12.	0.599^cdef^ ± 0.11	62.38^bc^ ± 8.51	88.11^j^ ± 1.11	0.440^f^ ± 0.04	4.87^g^ ± 0.13f	0.495^f^ ± 0.01	539.58^ef^ ± 4.5	1.28^a^ ± 0.27
13.	0.507^c^ ± 0.03	48.07^b^ ± 4.09	40.72^g^ ± 1.62	0.348^e^ ± 0.04	4.25^de^ ± 0.25	0.436^d^ ± 0.01	426.07^d^ ± 4.9	1.31^a^ ± 0.20
14.	0.665^efg^ ± 0.03	64.84^cd^ ± 8.02	28.33^ef^ ± 0.58	0.602^g^ ± 0.05	5.31^gh^ ± 0.64	0.340^c^ ± 0.02	567.77^f^ ± 8.5	1.29^a^ ± 0.03
15.	0.542^cd^ ± 0.04	48.41^b^ ± 0.86	24.87^cde^ ± 0.57	0.272^bc^ ± 0.02	5.13^gh^ ± 0.24	0.448^de^ ± 0.01	573.04^f^ ± 2.1	1.35^a^ ± 0.18
16.	0.351^b^ ± 0.05	58.16^bc^ ± 1.28	21.36^bcd^ ± 6.22	0.248^bcd^ ± 0.08	5.16^gh^ ± 0.23	0.408^d^ ± 0.02	522.18^e^ ± 3.8	2.23^b^ ± 0.16
17.	0.372^b^ ± 0.12	58.65^bc^ ± 1.06	25.37^de^ ± 0.54	0.295^cde^ ± 0.03	3.85^bcd^ ± 0.24	0.489^ef^ ± 0.01	537.70^ef^ ± 8.0	1.46^a^ ± 0.17
18.	1.004^h^ ± 0.31	47.79^b^ ± 2.26	39.71^g^ ± 0.63	0.967^h^ ± 0.08	3.23^a^ ± 0.22	0.607^h^ ± 0.03	279.08^b^ ± 8.9	1.59^a^ ± 0.34

Arithmetic means followed by the same letter within a column indicate no significant difference (*p* < 0.05) in Duncan test.

**Table 3:** Phenolic compounds in *Morus alba* L. samples (arithmetic mean ± standard deviation).

	Gallic Acid	Vanillic Acid	Caffeic Acid	Chlorogenic Acid	FERULIC ACID	*p*-Coumaric Acid	Rosmarinic Acid	Rutin	Apigenin	Quercetin
Hydromethanolic extracts										
	(µg/g)	(µg/g)	(µg/g)	(mg/g)	(µg/g)	(µg/g)	(µg/g)	(mg/g)	(µg/g)	(mg/g)
**1.**	276.96^c^±1.92	nd	119.23^abcd^ ± 7.25	6.91^def^ ± 0.27	168.79^fg^ ± 1.82	44.11^cde^ ± 12.15	164.71^a^ ± 6.58	8.83^cd^ ± 1.53	282.95^ab^ ± 3.12	1.98^abc^ ± 0.36
**2.**	326.73^d^±1.60	5.99^b^ ± 1.05	141.89^e^ ± 9.77	9.44^efgh^ ± 0.77	173.79^gh^ ± 0.23	46.47^def^ ± 2.77	173.72^a^ ± 1.43	12.35^d^ ± 2.10	286.86^ab^ ± 7.04	4.70^e^ ± 0.50
**3.**	51.72^a^±2.50	nd	225.54^g^ ± 3.59	8.53^defgh^ ± 3.33	263.60^i^ ± 8.63	69.95^g^ ± 8.18	291.38^b^ ± 0.81	5.35^c^ ± 1.25	296.63^ab^ ± 2.45	3.92^de^ ± 0.46
**4.**	203.54^b^±7.62	nd	106.40^a^ ± 3.39	11.76^gh^ ± 1.78	121.25^de^ ± 2.23	26.17^a^ ± 4.10	193.50^a^ ± 5.69	8.95^cd^ ± 4.33	290.37^ab^ ± 3.18	2.40^bcd^ ± 0.47
**5.**	346.20^d^±4.46	nd	357.23^h^ ± 2.62	5.64^bcde^ ± 0.54	128.17def ± 1.02	49.44^ef^ ± 2.65	nd	6.09^c^ ± 2.61	283.56^ab^ ± 1.23	0.74^ab^ ± 0.17
**6.**	357.20^d^±3.42	9.82^d^ ± 1.43	108.99^ab^ ± 6.08	2.37^abc^ ± 0.10	59.24^ab^ ± 1.20	33.09^abcd^ ± 10.34	130.64^a^ ± 1.96	1.83^b^ ± 2.48	295.62^ab^ ± 1.19	0.65^ab^ ± 0.06
**7.**	73.04^a^±6.98	6.21^b^ ± 4.49	127.67^de^ ± 13.23	11.31^fgh^ ± 1.03	128.27^def^ ± 2.23	41.04^bcde^ ± 8.83	nd	11.20^d^ ± 1.33	344.22^b^ ± 7.39	1.56^ab^ ± 0.54
**8.**	229.29^bc^±2.23	nd	224.29^g^ ± 10.25	11.36^fgh^ ± 5.62	166.46^fg^ ± 1.47	60.42^fg^ ± 9.08	nd	3.12^bc^ ± 1.87	315.83^a^ ± 2.14	4.09^de^ ± 0.98
**9.**	302.12^d^±1.49	nd	103.34^a^ ± 4.32	2.05^ab^ ± 0.22	49.26^ab^ ± 0.18	47.80^ef^ ± 16.25	nd	0.83^a^ ± 0.04	283.63^ab^ ± 1.29	0.38^a^ ± 0.01
**10.**	74.01^a^±1.63	nd	109.30^ab^ ± 8.97	1.02^a^ ± 0.06	40.07^a^ ± 0.09	23.95^a^ ± 1.20	nd	0.87^a^ ± 0.16	308.99^ab^ ± 0.37	0.11a ± 0.03
**11.**	215.22^b^±5.61	nd	176.07^f^ ± 4.82	17.51^i^ ± 5.92	111.67^cd^ ± 0.31	32.14^abc^ ± 6.69	346.65^b^ ± 3.89	0.83^a^ ± 0.19	289.56^ab^ ± 8.75	6.40^f^ ± 0.26
**12.**	77.44^a^±8.64	7.84^c^ ± 1.62	101.47^a^ ± 0.30	12.36^h^ ± 6.05	101.40^cd^ ± 3.16	48.14^ef^ ± 14.21	204.50^a^ ± 1.57	11.57^d^ ± 5.62	326.61^b^ ± 2.89	3.48^cde^ ± 2.80
**13.**	80.24^a^±4.34	7.09^c^ ± 3.12	106.52^a^ ± 8.13	4.38^abcd^ ± 0.99	86.42^bcd^ ± 1.67	28.15^ab^ ± 5.59	172.81^a^ ± 2.29	2.82^b^ ± 0.50	340.36^b^ ± 8.57	1.48^ab^ ± 0.20
**14.**	72.58^a^±3.37	2.93^a^ ± 4.98	105.92^a^ ± 1.77	7.22^defg^ ± 1.26	188.92^gh^ ± 3.06	55.12^ef^ ± 5.17	147.41^a^ ± 1.81	5.52^c^ ± 2.35	313.62^b^ ± 3.47	1.76^abc^ ± 0.58
**15.**	77.11^a^±1.16	8.63^cd^ ± 1.05	113.40^bcde^ ± 1.65	6.71^abcd^ ± 0.89	115.49^efg^ ± 1.41	25.50^a^ ± 6.47	209.28^a^ ± 3.65	8.68^cd^ ± 4.48	293.49^ab^ ± 1.37	2.31^ab^ ± 0.43
**16.**	67.77^a^±6.19	nd	126.59^abc^ ± 4.08	4.37^cdef^ ± 0.41	154.50^cde^ ± 0.83	26.30^a^ ± 3.39	348.45^b^ ± 4.81	4.03^c^ ± 0.37	323.61^ab^ ± 1.26	0.79^bcd^ ± 0.18
**17.**	240.26^bc^±2.54	3.32^a^ ± 2.00	136.20^de^ ± 4.25	8.20^defgh^ ± 3.46	76.81^abc^ ± 3.60	26.86^a^ ± 3.34	178.88^a^ ± 1.36	2.02b ± 0.97	288.97^ab^ ± 2.98	1.35^ab^ ± 0.33
**18.**	273.95^c^±5.30	nd	424.00^i^ ± 10.36	10.72^fgh^ ± 14.71	212.48^h^ ± 2.24	44.81^cde^ ± 3.59	290.47^b^ ± 3.31	17.06^e^ ± 2.01	283.18^ab^ ± 1.51	6.88^f^ ± 2.50
Water extracts										
	(mg/g)	(µg/g)	(mg/g)	(mg/g)	(mg/g)	(mg/g)	(mg/g)	(mg/g)	(mg/g)	(mg/g)
**1.**	11.26^bcd^ ± 3.76	198.63^a^ ± 1.72	0.94^abc^ ± 0.03	7.61^abc^ ± 6.23	1.43^bcd^ ± 1.08	0.29abc ± 0.03	7.28^ef^ ± 0.16	55.63^d^ ± 0.25	2.48^a^ ± 0.16	10.10^cde^ ± 0.26
**2.**	3.78^ab^ ± 0.16	335.61^ab^ ± 0.01	1.35^c^ ± 0.81	1.97^a^ ± 0.16	0.62^ab^ ± 0.58	1.06^ef^ ± 0.03	6.03^de^ ± 0.01	22.18^c^ ± 0.36	2.52^a^ ± 0.17	10.83^cdef^ ± 2.25
**3.**	1.99^a^ ± 0.21	742.27^ef^ ± 0.60	1.08^abc^ ± 0.43	37.96^de^ ± 6.92	1.66^bcd^ ± 0.19	0.44^abcd^ ± 0.34	3.07^abcd^ ± 3.71	13.07^b^ ± 0.98	2.37^a^ ± 0.01	13.13^ef^ ± 0.65
**4.**	8.44^abcd^ ± 1.70	508.98^bcd^ ± 0.04	1.22^abc^ ± 0.35	38.15^de^ ± 1.89	1.97^cd^ ± 0.36	0.79^cde^ ± 0.74	3.70^abcd^ ± 0.26	30.06^cd^ ± 8.40	2.37^a^ ± 0.01	10.77^cdef^ ± 1.69
**5.**	9.12^abcd^ ± 0.42	437.40^abcd^ ± 0.24	1.09^abc^ ± 0.20	14.48^abcde^ ± 3.31	1.57^bcd^ ± 0.24	0.73bcde ± 0.60	3.88a^bcd^ ± 0.10	25.63^c^ ± 5.52	2.35^a^ ± 0.05	8.93^cd^ ± 0.59
**6.**	14.37^cd^ ± 2.32	507.93^bcd^ ± 1.52	1.17^abc^ ± 0.55	30.07^abcde^ ± 2.04	2.17^d^ ± 0.97	0.79^cde^ ± 0.10	3.70^abcd^ ± 0.12	46.92^d^ ± 8.98	2.57^a^ ± 0.21	17.62^g^ ± 7.36
**7.**	6.42^abc^ ± 0.27	319.45^abcd^ ± 2.77	0.90^a^ ± 0.54	33.79^cde^ ± 3.60	0.77^ab^ ± 0.01	0.23^ab^ ± 0.17	1.90^ab^ ± 0.24	12.91^b^ ± 2.12	2.37^a^ ± 0.01	10.62^cde^ ± 0.41
**8.**	8.72^abcd^ ± 0.93	717.36^ef^ ± 0.02	0.91^a^ ± 0.42	3.43^a^ ± 0.36	1.84^cd^ ± 0.63	0.49^abcd^ ± 0.20	4.99^cde^ ± 0.70	22.91^c^ ± 1.48	2.58^a^ ± 0.20	5.16^ab^ ± 1.35
**9.**	9.90^abcd^ ± 1.44	228.45^a^ ± 2.02	1.02^abc^ ± 0.50	2.25^a^ ± 0.32	0.83^abc^ ± 0.32	0.58^abcde^ ± 0.87	4.64^bcde^ ± 0.33	36.85^cd^ ± 4.51	2.59^a^ ± 0.21	11.71^def^ ± 0.56
**10.**	13.64^cd^ ± 2.56	563.60^cde^ ± 2.57	1.16^abc^ ± 0.69	2.40^a^ ± 0.59	1.12^abcd^ ± 0.12	0.73^bcde^ ± 0.68	3.96abcd ± 0.39	33.24^cd^ ± 8.88	2.70^a^ ± 0.18	3.94^ab^ ± 2.00
**11.**	1.65^a^ ± 0.36	393.81^abcd^ ± 0.17	0.89^a^ ± 0.22	2.67^a^ ± 1.44	0.20^a^ ± 0.00	0.92^def^ ± 6.77	1.54^a^ ± 1.07	9.64^a^ ± 6.88	2.33^a^ ± 0.10	7.45^bc^ ± 0.58
**12.**	2.19^a^ ± 0.20	326.49^abcd^ ± 2.83	1.05^abc^ ± 0.27	3.72^a^ ± 0.55	1.77^bcd^ ± 0.64	0.54^abcd^ ± 0.22	9.88^f^ ± 1.20	13.00^b^ ± 0.11	2.34^a^ ± 0.02	5.58^ab^ ± 1.43
**13.**	7.89^abcd^ ± 0.50	365.53^abc^ ± 1.09	0.94^abc^ ± 0.44	11.66^a^ ± 1.10	0.62^ab^ ± 0.01	0.18^a^ ± 0.07	1.11^a^ ± 0.11	14.06^b^ ± 1.44	2.37^a^ ± 0.04	11.09^def^ ± 0.88
**14.**	9.30^abcd^ ± 1.68	294.78^a^ ± 0.58	1.34^bc^ ± 0.11	32.26^bcde^ ± 1.92	1.76^bcd^ ± 0.93	0.91^def^ ± 0.38	1.94^abc^±0.14	13.57^b^ ± 8.19	2.51^a^ ± 0.16	14.33f ± 1.08
**15.**	9.70^abcd^ ± 1.44	322.46^a^ ± 0.12	1.20^ab^ ± 0.72	5.13^abc^ ± 0.90	2.05^d^ ± 0.49	1.77^def^ ± 7.88	8.85^abcd^±7.34	23.80^c^ ± 1.21	2.59^a^ ± 0.08	4.70^g^ ± 0.13
**16.**	17.78^abcd^ ± 4.32	204.68^ab^ ± 1.77	0.92^abc^ ± 0.71	6.19^ab^ ± 1.11	1.68^bcd^ ± 1.08	0.77^cde^ ± 1.14	4.06^f^ ± 0.15	18.15^bc^ ± 4.71	2.46^a^ ± 0.19	20.05^h^ ± 6.98
**17.**	4.45^ab^ ± 1.73	642.82^f^ ± 5.57	0.90^a^ ± 0.67	2.33^a^ ± 0.39	1.16^abcd^ ± 0.68	0.45^abcd^ ± 0.57	2.95^abc^ ± 1.09	87.27^e^ ± 7.31	2.57^a^ ± 0.14	3.60^a^ ± 0.29
**18.**	16.08^d^ ± 1.43	593.01^de^ ± 1.61	1.77^d^ ± 0.24	41.18^e^ ± 3.92	1.65^bcd^ ± 0.82	1.30^f^ ± 0.21	1.16^a^ ± 0.27	26.51^c^ ± 6.17	3.12^b^ ± 0.28	23.51^h^ ± 2.20

Arithmetic means followed by the same letter within a column indicate no significant difference (*p* < 0.05) in Duncan test.nd: not detectable.

**Table 4:** Antioxidant activity and acetylcholinesterase (AChE) inhibitory activity of *Morus alba* L. samples.

	Hydromethanolic Extract		Water Extracts		
	DPPH mg TE/100 g)	FRAP (mmol Fe^2+^/g)	DPPH (mg TE/100 g)	FRAP (mmol Fe^2+^/g)	% AChE Inhibition
1.	27.63^bc^ ± 0.78	30.16^ij^ ± 0.56	55.64^c^ ± 2.56	7.40^ab^ ± 1.28	29.2 ± 3.32
2.	24.53^ab^ ± 0.29	27.38^h^ ± 0.28	76.72^h^ ± 3.04	6.53^ab^ ± 1.41	15.9 ± 5.15
3.	26.42^abc^ ± 1.39	30.86^ij^ ± 0.50	78.76^ij^ ± 11.72	7.47^ab^ ± 0.11	37.7 ± 6.59
4.	33.21^d^ ± 0.94	29.59^d^ ± 0.35	91.08^l^ ± 4.83	15.12^i^ ± 0.67	36.1 ± 4.07
5.	24.55^ab^ ± 3.47	18.10^i^ ± 3.64	65.30^f^ ± 2.84	8.13^bcd^ ± 0.47	2.80 ± 0.54
6.	23.38^bc^ ± 0.17	34.10^k^ ± 0.22	79.94^j^ ± 7.84	7.68^abc^ ± 0.18	37.0 ± 4.73
7.	24.91^ab^ ± 0.27	20.32^e^ ± 0.24	61.75^e^ ± 6.37	9.71^def^ ± 1.03	13.4 ± 1.23
8.	24.57^ab^ ± 1.15	27.35^h^ ± 0.11	59.65^d^ ± 2.73	9.24^cde^ ± 0.13	25.7 ± 2.64
9.	34.60^d^ ± 0.33	16.86^cd^ ± 0.31	78.01^hi^ ± 5.68	6.43^ab^ ± 0.43	18.9 ± 0.57
10.	35.74^d^ ± 5.08	14.91^ab^ ± 0.59	85.62^k^ ± 3.83	5.96^a^ ± 0.39	7.13 ± 1.87
11.	26.33^abc^ ± 1.81	31.64^j^ ± 0.36	66.39^f^ ± 6.25	10.46^efg^ ± 0.47	3.75 ± 0.74
12.	25.65^abc^ ± 1.44	24.60^g^ ± 0.46	60.09^d^ ± 7.70	16.24^i^ ± 1.37	30.5 ± 5.80
13.	25.64^abc^ ± 0.68	13.49^a^ ± 0.41	52.41^b^ ± 6.26	11.69^gh^ ± 0.52	2.64 ± 1.47
14.	28.70^c^ ± 0.38	24.55^g^ ± 0.19	98.82^m^ ± 1.52	12.46^h^ ± 0.33	24.5 ± 2.44
15.	26.72b^c^ ± 0.38	23.58^bc^ ± 0.29	47.79^a^ ± 2.05	11.20^gh^ ± 1.30	34.2 ± 1.86
16.	27.50^bc^ ± 0.38	25.03^g^ ± 0.19	77.99^hi^ ± 3.22	11.91^fgh^ ± 1.2	36.3 ± 1.34
17.	24.74^ab^ ± 0.87	22.72^f^ ± 0.48	69.76^g^ ± 5.71	16.28^i^ ± 2.0	32.5 ± 1.08
18.	27.48^bc^ ± 0.55	37.85^l^ ± 0.39	64.95^f^ ± 4.71	21.21^j^ ± 0.99	36.1 ± 4.72

Arithmetic means followed by the same letter within a column indicate no significant difference (*p* < 0.05) in Duncan test.

**Table 5:** List of analyzed commercial samples of *Morus alba* L.

No.	Sample Name on the Package	Composition	Confection	Place of Origin of Plant Material
1.	White mulberry (*Morus alba* L.), supplement dietary	leaf	bags	Poland
2.	White mulberry (*Morus alba* L.), supplement dietary	leaf	bags	Poland
3.	White mulberry (*Morus alba* L.), supplement dietary	leaf	bags	Poland
4.	White mulberry (*Morus alba* L.), supplement dietary	leaf	bags	Bulgaria
5.	White mulberry (*Morus alba* L.), supplement dietary	leaf	bags	Poland
6.	White mulberry (*Morus alba* L.), supplement dietary	leaf	bags	Poland
7.	Leaf of white mulberry–express tea, supplement dietary	leaf	bags	Poland
8.	White mulberry, ecological tea	leaf	bags	Poland
9.	Ecological white mulberry (*Morus alba* L.), 100% organic	fruits	loose	Turkey
10.	White mulberry (*Morus alba* L.)	fruits	loose	Turkey
11.	Leaf of white mulberry	leaf	loose	Poland
12.	White mulberry (*Morus alba* L.), herbal tea	leaf	loose	Poland
13.	Leaf of white mulberry, ecological tea	leaf	loose	Poland
14.	White mulberry, herbal tea	leaf	bags	Poland
15.	Leaf and fruits of white mulberry	Leaf (90%) and fruits (10%)	loose	Poland
16.	Leaf of white mulberry, dried herbs	leaf	bags	Poland
17.	White mulberry 100%, herbal tea	herb	loose	Poland
18.	White mulberry with Yerba Mate and lemon, express tea	leaf	bags	European Union
